# Learning Out of Hours: A Narrative Review of Balancing Responsibility, Supervision, and Fatigue in Junior Doctor Training

**DOI:** 10.7759/cureus.118217

**Published:** 2026-10-20

**Authors:** Rosamma Lenitte Thomas

**Affiliations:** 1 Trauma and Orthopaedics, University Hospital Crosshouse, Glasgow, GBR

**Keywords:** clinical autonomy, clinical supervision, experiential learning, fatigue, junior doctors, out-of-hours care, postgraduate medical education

## Abstract

Out-of-hours work is an integral component of postgraduate medical training and exposes junior doctors to clinical responsibility, uncertainty, competing demands, and acute deterioration in circumstances that may differ substantially from routine daytime practice. These conditions can provide valuable opportunities for experiential learning, but they can also reduce educational value when responsibility is accompanied by inadequate supervision, excessive workload or fatigue. This narrative review examines out-of-hours work as a learning environment for junior doctors, integrating literature on workplace learning, autonomy and supervision, prioritisation and decision-making, deterioration and escalation, handover and teamwork, sleep disruption and fatigue, and educational interventions designed to prepare trainees for out-of-hours practice.

The literature suggests that the educational value of out-of-hours work arises not from independence alone, but from meaningful clinical responsibility within an appropriately supported environment. Responsibility can provide opportunities for autonomous initial assessment, prioritisation, and decision-making under uncertainty, while the recognition of personal limits and appropriate escalation are themselves components of developing clinical judgement. Simulation and structured preparation may help trainees rehearse these demands before substantial clinical responsibility is assumed, while feedback, reflection, and review of subsequent patient outcomes can help convert clinical exposure into learning. However, excessive workload, inadequate supervision, and fatigue may impair both the opportunity and the capacity to learn.

Out-of-hours training should therefore aim to preserve authentic responsibility while ensuring that autonomy is graded to capability, senior support remains accessible, workload is manageable, and opportunities for feedback and recovery are maintained. We propose that effective out-of-hours learning occurs within a zone of meaningful responsibility in which trainees are sufficiently challenged to develop clinical judgement without becoming unsupported or physiologically unsustainable. Designing this environment requires attention not only to individual preparedness but also to supervision, staffing, workload, handover, feedback, and rota design.

## Introduction and background

Out-of-hours (OOH) work is a longstanding component of postgraduate medical training. In this review, OOH work refers to clinical duties undertaken outside routine daytime working hours, principally during evenings, nights, and weekends, including on-call and night-shift responsibilities. For junior doctors, evenings, nights, and weekends can differ substantially from routine daytime practice: staffing may be reduced, senior clinicians may be less immediately visible, and responsibility may extend across unfamiliar patients and clinical areas. Work may involve responding to acute deterioration, managing competing tasks, interpreting handovers, communicating with nursing staff and other specialties, and deciding when senior assistance is required. A recent scoping review of 49 studies examining junior medical officers working OOH identified variable workloads, reduced training quality, challenges relating to supervision and wellbeing, and implications for patient safety, illustrating that OOH work represents both a clinical service requirement and a distinctive working environment for doctors early in training [1].

Clinical responsibility refers here to the tasks and decisions a junior doctor is expected to undertake, while autonomy describes the degree of independence exercised in carrying them out. Supervision refers to the availability and involvement of more experienced clinicians in guiding, supporting, and reviewing those decisions; it does not necessarily require their continuous physical presence [2-4]. Fatigue refers to reduced capacity associated with sustained work, inadequate sleep, or disrupted sleep-wake patterns, which may affect clinical performance and engagement with learning [5-7].

The conceptual approach adopted in this review draws on established perspectives in medical education rather than proposing a new theory of learning. Experiential and workplace-learning literature emphasises that professional development occurs through participation in authentic clinical activities, with opportunities for feedback and support shaping how experience contributes to learning [8,9]. Progressive independence and supervisory entrustment provide a complementary perspective: clinical responsibility should develop in relation to a trainee's capability, while supervision remains responsive to the clinical context [2-4,10]. These perspectives provide the educational foundation for examining how responsibility, autonomy, and supervision interact during OOH work. The proposed zone of meaningful responsibility extends this synthesis by considering how workload, fatigue, and the organisational conditions of OOH practice may support or disrupt these learning processes.

These characteristics may also create important opportunities for workplace learning by requiring junior doctors to apply knowledge in authentic clinical situations. However, clinical exposure should not automatically be equated with educational value: excessive work pressure may disrupt learning, while supervision, feedback, and the wider practice environment influence whether experience becomes educationally productive [9]. OOH work can therefore require junior doctors to move from observing or discussing clinical decisions to performing initial assessments, establishing priorities, formulating management plans, and determining when further expertise is required.

Evidence concerning on-call learning illustrates this distinction. In a recent qualitative study, Baten et al. found that learning during on-call work was associated with responsibility, uncertainty, independent judgement, and participation in professional practice, while supervisors shaped learning through calibrated autonomy, accessible guidance, and feedback [11]. These findings suggest that the educational value of OOH work does not arise simply because junior doctors work with less direct supervision; rather, responsibility can create opportunities for autonomous clinical reasoning when appropriate support remains available.

Preparation for these demands is itself an educational challenge. A recent UK scoping review identified 18 studies evaluating interventions designed to prepare medical students for OOH practice, predominantly using simulation and addressing competencies including prioritisation, communication, clinical decision-making, and leadership. Improvements in confidence or attitudes were reported more often than objective performance or behavioural outcomes, and longer-term follow-up was limited [12]. OOH preparedness therefore involves more than mastery of isolated clinical tasks: trainees must learn to manage responsibility, uncertainty, interruptions, prioritisation, and communication within the organisational realities of clinical practice.

The same features that create learning opportunities may, however, impose educational and clinical costs. Responsibility may promote autonomy, but autonomy without accessible supervision can become unsupported practice. Competing demands may develop prioritisation, but excessive workload may produce cognitive overload. Clinical challenges may promote development, but sleep disruption and fatigue can impair the attention and decision-making on which both learning and safe care depend. Recent OOH literature similarly identifies reduced training quality and diminished junior-doctor wellbeing, alongside challenges associated with workload, staffing, and supervision [1]. The relevant educational question is therefore not whether OOH work is intrinsically beneficial or harmful, but under what conditions its challenges remain developmentally useful rather than becoming unsupported or physiologically unsustainable.

This narrative review examines OOH work as a learning environment for junior doctors through five interconnected domains: experiential workplace learning; the development of autonomy, prioritisation, and decision-making; recognition of deterioration, escalation, and multidisciplinary working; the educational consequences of workload, sleep disruption, and fatigue; and strategies for creating safer and more effective OOH learning environments. Previous literature has examined night-float education, OOH workload and safety, learning during on-call work, and preparation for OOH practice from different perspectives [1,11-13]. However, these findings have received less integrated consideration in relation to how clinical responsibility becomes educationally productive and how supervision, organisational demands, and fatigue may support or disrupt that process. The present review therefore brings these related literatures together to examine progressive autonomy, accessible supervision, workload, and fatigue as interconnected features of the OOH learning environment.

The central question guiding this narrative review is: Under what conditions does OOH clinical responsibility support learning and professional development in junior doctors, and when might it become educationally or clinically counterproductive? The aim of this review is to synthesise evidence concerning experiential learning, clinical autonomy, supervision, deterioration and escalation, workload and fatigue, and educational interventions in OOH practice, and to propose a conceptual framework describing the conditions under which meaningful clinical responsibility may support junior doctors' learning.

This review proposes that meaningful clinical responsibility can support the development of autonomous assessment, prioritisation, and decision-making when matched to trainees' capabilities and supported by accessible supervision [2-4,10]. Recognition of personal limits, appropriate escalation, collaborative management, feedback, and reflection are also important to this learning process [4,9,11]. The proposed framework considers how these educational opportunities interact with workload, fatigue, and the organisational conditions of OOH practice [1,5-7], with the aim of identifying principles for supporting responsibility without compromising learning or safety.

Literature searching for this narrative review began in approximately late July to early August 2026 and continued through September 2026. Initial targeted searches were conducted using PubMed and relevant medical and medical-education journal and publisher sources to identify literature on OOH work and postgraduate medical training. Search concepts included combinations of terms such as "out-of-hours", "on-call", "night shift", "night float", "junior doctor", "resident", "medical education", "clinical responsibility", "autonomy", and "supervision". As the manuscript developed, further topic-specific searches were undertaken to inform each of the five review domains, including clinical decision-making, deterioration and escalation, teamwork, workload, fatigue, feedback, and educational interventions. Relevant primary studies, reviews, and professional guidance were selected for their contribution to the review's themes, with attention to the applicability of findings across different training and healthcare contexts. References were organised using Zotero, and a literature spreadsheet was used to record relevant findings, consider their relevance to the review themes, and note limitations identified during reading. The evidence was integrated thematically to examine relationships between responsibility, supervision, learning opportunities, and the organisational and physiological demands of OOH work. The search and selection process was iterative and relevance-based rather than systematic; a predefined search protocol, exhaustive screening procedure, and formal risk-of-bias assessment were not undertaken.

Given the narrative scope of this review and the heterogeneity of the included literature in study design, setting, and outcome measures, findings were synthesised qualitatively rather than statistically pooled. No meta-analysis, meta-regression, or other statistical modelling was undertaken.

## Review

OOH work as an experiential learning environment

Learning Through Clinical Work and Responsibility

The educational significance of OOH work can be understood through experiential and workplace-learning perspectives, which emphasise the application of knowledge through authentic clinical participation [8,9]. For junior doctors, clinical responsibility may create opportunities to move beyond observing decisions towards assessing problems, formulating initial plans, and evaluating their clinical judgement. However, the relationship between service and education is not automatic; excessive work pressure may disrupt rather than facilitate learning [9].

OOH work provides a distinctive setting in which such experiential learning can occur. Compared with routine daytime practice, junior doctors may encounter reduced staffing, fewer immediately available members of the usual clinical team, unfamiliar patients, competing demands, and a greater need to determine which problems require immediate attention [1]. Rather than reproducing the structured teaching environment of daytime practice, learning may arise directly from patient care: assessing a new problem, developing an initial management plan, responding to changing clinical information, seeking appropriate advice, and reflecting on the outcome of decisions.

Recent qualitative evidence supports this interpretation. Baten et al. found that learning during on-call work extended beyond clinical competence to participation in professional practice and independent judgement, with responsibility and uncertainty acting as important features of the learning process [11]. Organisational and supervisory conditions shaped whether those experiences became useful learning opportunities.

The educational significance of this environment may therefore lie partly in the transition from knowing what should be done to deciding what to do in practice. In routine daytime work, junior doctors can observe senior decision-making, participate in ward rounds, and develop clinical knowledge within relatively well-supported teams. OOH work can require this knowledge to be applied in circumstances in which the junior doctor must first assess the problem and formulate an initial response before deciding whether senior input is required. This should not be interpreted as an argument for unsupported practice. Rather, it illustrates how responsibility, when appropriately supervised, can transform clinical service into a learning experience.

Learning to Work With Competing Clinical Demands

An important characteristic of OOH practice is that clinical problems rarely arrive in a convenient sequence. Junior doctors may simultaneously receive requests concerning deteriorating patients, medication or prescribing problems, investigation results, new admissions, and tasks handed over by daytime teams. Consequently, the educational experience is not limited to learning the management of individual diseases. It also involves learning how to organise clinical work when not every task can receive immediate attention.

Simulation studies attempting to reproduce on-call work provide useful evidence about these demands. Manalayil et al. developed an on-call simulation for final-year medical students in which participants received multiple bleeps and deliberately more tasks than could realistically be completed within the allotted period. The exercise required participants to obtain relevant information, distinguish urgency, communicate with other healthcare professionals, and prioritise competing tasks; senior advice remained available when required. Participants reported increased confidence, and the authors identified resourcefulness, communication, and triage as important components of preparedness for on-call practice [14].

Similar principles were incorporated into an immersive on-call simulation for newly appointed UK Foundation Year 1 doctors. Participants received a clinical handover and undertook realistic tasks while having access to a simulated registrar, followed by structured debriefing. The programme was designed specifically to allow new doctors to practise prioritising their workload and managing the technical and non-technical aspects of on-call work before encountering them independently in clinical practice [15].

These findings also illustrate why effective handover and prioritisation are closely related during OOH work. A doctor covering several clinical areas cannot treat every outstanding daytime task as equally urgent. Handover should therefore support continuity of care while allowing the receiving doctor to identify patients and tasks requiring action during the OOH period. Determining what requires immediate review, what can safely wait, what information is needed to make that judgement, and when further help should be sought are themselves components of clinical reasoning. The detailed roles of handover, escalation, and interprofessional communication are considered later in this review.

These interventions are consistent with Gordon et al.'s UK scoping review of OOH preparedness, in which simulation predominated and prioritisation, communication, clinical decision-making, and leadership were recurring targets. The review also highlighted the relative scarcity of objective performance, behavioural and longer-term outcomes compared with confidence-based measures [12].

From Repeated Exposure to Preparedness and Confidence

The educational value of OOH work may also accumulate through repeated exposure. Clinical situations that initially produce uncertainty can become more manageable as junior doctors acquire familiarity with hospital systems, recognise patterns of deterioration, develop approaches to common problems, and become more confident about the limits of their own competence. Importantly, increasing confidence should not be equated with increasing willingness to manage patients without assistance; appropriate preparedness includes recognising when escalation is necessary.

This distinction is supported by research involving UK Foundation Year 1 doctors. In a qualitative study of seven FY1 doctors, Burridge et al. found that participants initially described responding to acutely unwell patients as overwhelming and challenging, with uncertainty regarding when to escalate and concerns about being expected to act beyond their competence. After approximately three to six months of clinical work, participants reported greater preparedness. Hands-on experience, reflection, simulation, and multidisciplinary team working were consistently regarded as particularly useful learning experiences. Preparedness included familiarity with initial management, recognition of clinical severity, understanding the hospital environment, and knowing when senior support was required [16].

Evidence from night-float programmes provides a similarly nuanced picture. In a survey of internal medicine residents, night-float rotations were rated negatively overall as educational experiences, with residents reporting less interaction with attending physicians, fewer formal educational activities, and disruption to sleep and personal life. Nevertheless, residents identified autonomy and opportunities to develop triage skills as positive aspects of the rotation [17]. Other work has likewise demonstrated that residents and faculty perceive fewer traditional teaching and learning opportunities at night than during daytime rotations [18]. These findings are important because they distinguish teaching from learning: an environment may contain less formal teaching while still generating significant workplace learning.

More recent qualitative research makes this distinction particularly clear. In a study involving 12 family medicine and paediatric interns at a US tertiary children's hospital, Torwekar et al. found that participants described rich experiential learning during night-float rotations and did not necessarily want nighttime education to replicate daytime didactic teaching. Instead, they valued opportunities to initiate patient assessments independently, receive informal teaching prompted by actual patient care, know that supervisory support was accessible, and obtain feedback on their decisions [19]. This model closely reflects the potential educational role of OOH work: the junior doctor encounters and initially engages with a clinical problem, while senior support remains available rather than absent.

The educational value of OOH work should nevertheless be interpreted cautiously. Studies describing greater confidence, preparedness, or perceived learning often rely on trainees' self-reported experiences, which do not necessarily establish objectively measured improvements in clinical performance [11,16,19]. Conversely, studies of night-float rotations have identified reduced formal teaching opportunities, less interaction with attending physicians, and disruption to sleep, despite trainees also recognising opportunities to develop autonomy and prioritisation skills [17,18]. Reviews of educational interventions and night-float arrangements likewise highlight variation in educational approaches and limitations in the evidence for sustained improvements in performance [12,13]. These findings suggest that neither exposure to night work nor the amount of clinical responsibility undertaken can, by itself, be assumed to produce educational benefit.

Developing clinical autonomy, prioritisation, and decision-making

Developing Autonomy Through Progressive Responsibility

Autonomy is a central but sometimes misunderstood component of postgraduate medical training. Clinical autonomy does not imply practising without supervision; rather, it describes the progressive assumption of responsibility as trainees develop the knowledge, skills, and judgement required for increasingly independent clinical practice. Progressive independence has long been regarded as an important principle of clinical training, although the empirical evidence defining how autonomy should optimally be provided remains limited [2].

The educational importance of autonomy has been examined more systematically in recent literature. In a scoping review of 189 publications, Allen et al. found that only 14 specifically investigated the educational role of autonomy, while much of the remaining literature reflected the perceptions or opinions of trainees, supervisors, or authors. Across the literature, autonomy was associated with outcomes including confidence, readiness for practice, clinical decision-making, and professional identity formation [3]. These associations should not be interpreted as evidence that increasing autonomy necessarily produces better educational outcomes. Rather, they indicate that opportunities to exercise responsibility are repeatedly perceived as important components of professional development.

The challenge is therefore not simply to maximise autonomy, but to calibrate it appropriately. In a national multispecialty panel of residents and fellows, Franzone et al. identified graduated responsibility, individual assessment, mentorship, and two-way communication as important components of progressive independence [10]. Similarly, a recent scoping review by Shim et al. found that supervisory entrustment influenced trainees' experiences of autonomy, confidence, responsibility, uncertainty, learning goals, and professional identity. Both excessive and insufficient supervision could adversely affect learning and professional development, reinforcing the importance of matching autonomy to the individual trainee's capability and clinical context [4].

Prioritisation and Decision-Making Under Competing Demands

Autonomy during OOH work is closely connected to prioritisation. A junior doctor responsible for several wards or clinical areas cannot respond to every request simultaneously and must determine which patients require immediate assessment, which tasks can safely wait, and which problems require escalation. This involves more than just organisational efficiency. Prioritisation requires the integration of clinical information, assessment of urgency and potential risk, allocation of limited time and attention, and repeated reassessment as new information or requests emerge.

Evidence from simulation demonstrates that prioritisation can be deliberately practised. Seale et al. compared 25 final-year medical students who received training in telephone communication and prioritisation with 25 untrained students. Trained students obtained more task-relevant information, prioritised clinical job lists more successfully, and reported greater confidence [20]. Together with OOH simulation and preparedness literature [12,14], this supports prioritisation as a skill that can be taught and rehearsed rather than left entirely to incidental exposure.

Prioritisation also depends on recognising uncertainty. The junior doctor may initially have incomplete information and may need to act before a diagnosis is established or all investigations are available. Clinical judgement therefore includes deciding what additional information is required, how urgent the problem is, and when uncertainty itself should prompt senior input.

Balancing Autonomy With Supervision

The educational value of autonomy depends heavily on the way supervision is provided. If supervisors intervene in every decision before a trainee has an opportunity to assess the problem independently, opportunities to develop clinical judgement may be reduced. Conversely, if supervision is inaccessible or responsibility exceeds the trainee's competence, autonomy can become unsupported practice. The relevant distinction is therefore not between supervision and autonomy but between forms of supervision that enable progressive responsibility and those that either restrict or inadequately support it [2-4].

Evidence from OOH settings suggests that increased senior availability does not necessarily eliminate trainee autonomy. Haber et al. examined the introduction of an in-hospital overnight attending physician and found improvements in residents' perceptions of the adequacy of supervision and the clinical value of the rotation, together with greater attending involvement in important clinical decisions. Importantly, these changes occurred without a statistically significant reduction in residents' perceived decision-making autonomy [21]. This suggests that senior availability is not equivalent to senior intrusion: supervision can be readily accessible while still allowing junior doctors to undertake initial assessment and clinical reasoning.

Evidence from daytime inpatient practice provides an important qualification. In a randomised crossover trial of increased attending supervision on a daytime general medical service, Finn et al. found no statistically significant reduction in medical-error rates with increased direct supervision. Interns spoke less during rounds, while residents reported lower perceived autonomy and less improvement in comfort making independent decisions when attendings joined work rounds [22]. Although this study concerned daytime inpatient supervision rather than OOH work, it illustrates the potential educational consequences of supervision that becomes more directly intrusive.

Taken together, these findings suggest that the educational effects of supervision depend not simply on its availability or intensity, but also on how it is provided and the clinical context [21,22]. Greater overnight attending availability may improve perceived supervision without necessarily reducing trainees' perceived decision-making autonomy, whereas more direct attending involvement during daytime rounds may constrain opportunities for independent judgement [21,22]. Equally, independence resulting from limited access to senior support should not automatically be regarded as beneficial autonomy, particularly when responsibility exceeds a trainee's competence [2,4]. Within the conceptual framework proposed in this review, graded autonomy and accessible supervision therefore serve complementary functions: junior doctors need opportunities to undertake initial assessments, establish priorities, and formulate decisions appropriate to their developing capability, while senior expertise remains reliably accessible when clinical severity, uncertainty, or competence limits require it [2,4,10]. Effective supervision need not involve continuous physical presence; appropriate entrustment, timely assistance, and opportunities to discuss decisions and receive feedback are also important [4,11,21]. This interpretation supports progressive responsibility rather than either maximal independence or constant senior intervention, although the available evidence does not establish an optimal level of supervision.

Recognising that a situation exceeds one's current competence and seeking appropriate assistance is itself an exercise of clinical judgement. This connection between autonomy and escalation becomes especially important when managing deteriorating patients and is considered in the next section.

Managing deterioration, escalation, and working with the OOH team

Recognising and Responding to Clinical Deterioration

The recognition and initial management of clinical deterioration represent important responsibilities for junior doctors working OOH. On hospital wards, nurses frequently identify changes in a patient's condition and request medical review, with the on-call junior doctor often being among the first medical practitioners to assess the patient. An integrative review by Callaghan et al. identified 33 studies examining factors influencing junior doctors' ability to recognise, respond to, and manage deteriorating patients, 18 of which were conducted in the United Kingdom. The review found that junior doctors' capacity to manage deterioration was influenced by clinical experiential exposure, educational approaches incorporating non-technical skills, simulation, and the level and type of supervision available within the clinical environment [23].

Managing deterioration requires more than recognising an abnormal physiological parameter. The junior doctor must assess the patient systematically, interpret clinical information, identify immediately life-threatening problems, initiate appropriate management, assess the response to treatment, and determine whether additional expertise is required. The Resuscitation Council UK's ABCDE approach reflects this process, emphasising simultaneous assessment and treatment, repeated reassessment, early recognition of the need for additional help, the use of the wider healthcare team, and structured communication [24]. These activities combine technical knowledge with prioritisation, communication, and decision-making under uncertainty.

For doctors early in postgraduate training, competence in these situations develops progressively rather than being fully established at the beginning of practice. In a qualitative study of seven UK Foundation Year 1 doctors, Burridge et al. found that newly qualified doctors initially described managing acutely unwell patients as challenging and sometimes overwhelming. Difficulties included obtaining and interpreting relevant information, establishing priorities, and determining when senior support was required. Greater preparedness developed through clinical experience, reflection, multidisciplinary working, and educational interventions including simulation [16].

Educational research supports the role of structured preparation alongside workplace exposure. A systematic review by Connell et al. identified 23 studies evaluating educational interventions for recognising and managing deteriorating patients. Most programmes reported positive effects on learner outcomes, organisational systems, or patient-related outcomes, although study designs and outcome measures varied considerably. Simulation featured prominently, with all but one programme using blended teaching and more than 87% incorporating medium- or high-fidelity simulation [25]. A subsequent BEME systematic review specifically examining medical students and doctors in training identified 22 studies meeting its final criteria and found a wide range of educational approaches, including simulation, team-based training, virtual environments, and blended learning [26].

Simulation and workplace experience should therefore be viewed as complementary rather than competing approaches. Simulation can provide opportunities to practise recognition, initial management, communication, and escalation before these skills are required during genuine emergencies. Clinical practice then introduces uncertainty, competing demands, and responsibility that cannot be completely reproduced in a simulated environment. OOH work may consequently become an important setting in which previously acquired knowledge is applied to authentic clinical deterioration, provided that appropriate senior support remains accessible.

Knowing One's Limits and Escalating Appropriately

A central component of managing deterioration is recognising when the patient's needs exceed the junior doctor's current competence or the resources immediately available. Escalation is therefore part of clinical judgement: the doctor assesses the patient, initiates appropriate immediate management, recognises unresolved risk, and seeks more experienced assistance when required.

This principle is explicit in professional and clinical guidance. The Resuscitation Council UK advises clinicians managing deteriorating patients to recognise when additional assistance is required and to call for appropriate help early [24]. Current General Medical Council guidance similarly states that doctors accepting delegated responsibilities must prioritise patient safety and seek help or request that a more experienced colleague take over if they become concerned that the task cannot be performed safely [27]. Safe autonomy, therefore, includes awareness of the limits of one's competence and the ability to act on that awareness.

However, escalation is influenced by more than clinical severity alone. Rotella et al. studied 50 junior medical officers and found that decisions to escalate were influenced by confidence and familiarity with the cause of deterioration. Although only 12% reported limiting escalation because of fear of criticism or conflict, 36% expressed concern about waking senior colleagues overnight. Participants identified clear handover, documented treatment goals, and plans for anticipated deterioration as important ways to improve escalation [28]. These findings demonstrate how OOH decision-making can be influenced by interpersonal and organisational considerations in addition to clinical knowledge.

This is particularly relevant OOH, when supervision may be less continuously visible. Tucker et al. noted that junior doctors may need to seek supervision actively during OOH work, making recognition of personal limits and timely access to senior assistance important features of safe practice [1].

The relationship between junior doctors and other healthcare professionals is similarly important. In a qualitative study involving 10 junior doctors and 14 registered nurses at a Singapore acute general hospital, Chua et al. found that decisions concerning escalation of deteriorating ward patients were influenced not only by the severity of deterioration and perceptions of the medical team's ability to manage it, but also by professional hierarchy and sociocultural factors [29]. Consequently, the ability to escalate safely includes communicating concerns clearly, responding constructively to concerns raised by colleagues, and working within a culture in which seeking assistance is treated as appropriate clinical behaviour rather than evidence of failure.

Experience should therefore make junior doctors better at determining when senior involvement is necessary, communicating the reason for escalation and contributing to subsequent management. Senior involvement can also become a learning opportunity when the initial assessment is discussed and feedback is provided, allowing escalation to complete rather than interrupt the learning cycle.

Communication, Handover, and the OOH Team

OOH practice is inherently collaborative. Although a junior doctor may perform the initial medical assessment, deteriorating patients are rarely managed by one clinician in isolation. Nurses may first recognise deterioration and provide important information about changes from baseline; senior doctors contribute additional clinical expertise and decision-making; other specialties, critical care teams and allied healthcare professionals may become involved as the patient's needs evolve. Learning to work effectively within this network is therefore an important component of OOH professional development.

Communication is particularly important when urgency is high and information is incomplete. The Resuscitation Council UK recommends using the whole clinical team during the assessment of a deteriorating patient so that interventions can occur simultaneously and advocates structured communication approaches such as SBAR or RSVP [24]. For junior doctors, these situations can provide early opportunities to develop non-technical skills: communicating urgency, allocating immediate tasks, synthesising information for senior colleagues, and maintaining situational awareness while treatment is underway.

These behaviours also represent an early form of clinical leadership. Leadership in this context does not require the most junior doctor to possess the greatest expertise or to control every aspect of care. Rather, it can involve recognising a problem, initiating an organised response, ensuring that relevant team members are involved and transferring leadership appropriately when a clinician with greater expertise arrives. Effective teamwork therefore complements rather than diminishes individual responsibility.

Handover extends these principles across shifts and clinical teams. The Royal College of Physicians defines handover as the transfer of responsibility for immediate and ongoing care between healthcare professionals and specifically recognises nights, weekends and emergency admissions as periods when responsibility may pass between teams. Its guidance recommends standardised handover processes, written information combined with verbal communication where appropriate, training in prioritising patients and work, and particular attention to unstable or unwell patients. The sickest patients may require bedside handover with senior medical involvement [30].

Handover is therefore more than the transfer of a list of outstanding jobs. Effective OOH handover should allow the receiving clinician to understand which patients are at greatest risk, what has already occurred, what requires action during the shift and what response is expected if the clinical situation changes. Rotella et al.'s finding that junior doctors regarded clear handover with documented treatment goals and contingency plans as an important means of improving escalation further demonstrates the connection between handover and subsequent clinical decision-making [28].

Current GMC guidance reinforces this responsibility by requiring doctors to ensure that information necessary for ongoing care has been shared before going off duty and, where practical, that a named clinician or team has taken over responsibility when their involvement ends [31]. This is especially relevant OOH, when continuity frequently depends on clinicians caring for patients whom they have not previously met.

OOH learning therefore extends beyond independent problem-solving. Junior doctors must recognise deterioration, initiate management, understand the limits of their competence, escalate concerns, communicate effectively and work within a multidisciplinary team. Independence and collaboration are complementary: progressive autonomy includes knowing when to act and when patient safety requires the expertise of others.

The educational cost of working OOH

Sleep Disruption, Circadian Misalignment, and Fatigue

Night work requires doctors to remain clinically active at a time when they would ordinarily be sleeping. Consequently, the educational opportunities created by OOH work cannot be separated from the effects of sleep disruption and fatigue. These effects are particularly relevant to junior doctors because many of the capabilities expected during an on-call shift - including sustained attention, prioritisation, clinical reasoning, communication and recognition of deterioration - depend on maintaining effective cognitive performance throughout the working period.

UK research demonstrates that fatigue during OOH work is not determined by scheduled hours alone. Morrow et al. explored the experiences of 82 doctors from nine UK deaneries, including 53 foundation trainees and 29 specialty trainees, following the introduction of restricted working hours. Participants continued to describe significant fatigue and identified workload intensity, unpredictable working patterns, insufficient rest, staff shortages and working beyond scheduled hours as contributing factors. Fatigue was perceived to affect judgement, efficiency, mood and the capacity to learn [5]. These findings are important educationally because reducing nominal working hours does not necessarily ensure that the hours actually worked provide a sustainable learning environment.

Similar concerns were identified in a survey of 524 junior doctors in North West England. Among the 462 respondents who answered the relevant questions, 37% reported that they never obtained a natural break during night shifts, while 53% reported that they never achieved the recommended 20-45-minute nap [32]. Demanding workload, inadequate rest facilities and concerns regarding personal and patient safety featured prominently in respondents' experiences [32]. Such findings demonstrate that fatigue is partly physiological but is also shaped by the organisation of the workplace: opportunities for rest, workload and the design of shift patterns influence whether night work becomes tolerable or excessively fatiguing.

Shift design itself may therefore determine part of the educational cost of OOH practice. Brown et al. found that junior doctors recognised positive aspects of working on call and at night, including hands-on experience in clinical situations requiring critical judgement and confident decision-making, while simultaneously describing negative effects of demanding shift patterns on fatigue, training and work-life balance. Poorly designed schedules could reduce opportunities and motivation for learning and leave doctors with insufficient time or energy for educational activity. Conversely, workplace social support could partly buffer the adverse effects of arduous schedules and intense workload [6].

The educational value of responsibility therefore depends partly on the learner's physiological capacity to engage with it. When fatigue substantially impairs attention and cognitive performance, additional clinical exposure may provide diminishing educational benefit while increasing clinical risk.

When Workload and Fatigue Impair Learning and Wellbeing

OOH work combines altered sleep with workload, interruptions, competing demands and reduced access to the full daytime workforce. Some challenge is necessary for experiential learning, but educational challenge and excessive cognitive burden are not equivalent.

Taken together, the findings of Morrow et al. and Brown et al. suggest two related educational consequences of fatigue and demanding work patterns: opportunities for learning may be reduced, while trainees' capacity to engage with the opportunities that remain may also be impaired [5,6]. This distinction is important because clinical exposure can continue even when the conditions required to learn effectively from that exposure are compromised.

This relationship is unlikely to depend solely on the duration of an individual shift. Tucker et al. studied 336 junior doctors and found that fatigue and wellbeing were associated with features of work scheduling beyond total working hours, including the number of consecutive night shifts, recovery time following nights, frequency of on-call work and short intervals between shifts [7]. Thus, two shifts of identical duration may represent substantially different physiological and educational experiences.

Tucker et al.'s more recent scoping review similarly identified variation in OOH workload, reduced training quality and diminished wellbeing, with workload and supervision emerging as important features of the working environment [1]. Together with evidence concerning consecutive nights and recovery intervals [7], these findings suggest that educational and physiological demands depend on how OOH work is organised, not simply on shift duration.

The consequences extend beyond immediate tiredness. Repeated disruption to sleep and recovery can affect work-life balance and wellbeing, while demanding shift patterns may interfere with educational activities. Brown et al. identified work-life balance and social support as important dimensions of junior doctors' experiences, with supportive workplace relationships partly buffering the effects of arduous schedules [6]. More broadly, a systematic review and meta-analysis of nine randomised trials found that shorter resident duty hours were associated with modest reductions in emotional exhaustion and dissatisfaction with overall wellbeing compared with longer hours [33].

Fatigue is therefore not solely a wellbeing issue. It intersects directly with the capabilities OOH work is expected to develop: prioritisation requires sustained attention, deterioration recognition requires vigilance, decision-making requires integration of information, and handover and escalation require accurate communication and awareness of uncertainty.

Insufficient supervision can compound these pressures. Uncertainty may stimulate clinical reasoning when workload is manageable and senior advice is accessible, but the same uncertainty may become unsafe when fatigue, excessive workload, and inadequate support coincide. The relevant distinction is between supported challenge and excessive demand.

Although fatigue and high workload are consistently identified as concerns in OOH practice, their effects on educational outcomes are difficult to isolate from other organisational factors [5-7]. Working-time restrictions may reduce some exposures associated with fatigue while also altering continuity, clinical exposure and opportunities for learning [33,34]. Consequently, findings from studies of duty-hour reform should not be interpreted as demonstrating that shorter or longer shifts necessarily produce better education. Differences in study design, outcome measures, specialty, and healthcare setting limit direct comparison and support a cautious interpretation of the evidence [33,34].

From Educational Burden to Patient and Clinician Safety

The potential effects of fatigue become particularly important when educational considerations intersect with patient safety. Landmark studies of extended-duration work demonstrated that prolonged working periods can have measurable consequences. In a prospective randomised study of interns working in intensive care units, Landrigan et al. found 35.9% more serious medical errors during a traditional schedule containing frequent shifts of 24 hours or longer than during an intervention schedule that eliminated extended-duration shifts and reduced weekly working hours [35]. Barger et al. subsequently reported increased risks of motor-vehicle crashes and near-miss incidents following extended-duration shifts among interns [36]. These studies provide important evidence linking extended-duration work with safety outcomes, although their schedules and US training context should not be assumed to represent contemporary UK OOH practice directly.

The subsequent literature has produced a more complex picture. Restricting duty hours may reduce some forms of fatigue, but shortening working time can also alter continuity, clinical exposure and patterns of handover. Bolster and Rourke's systematic review of 27 studies examining duty-hour restrictions found that the most frequent findings were no improvement in patient care or resident wellbeing, while 64% of studies assessing educational outcomes reported an unfavourable educational effect. Night-float systems were the most frequently studied mechanism for implementing restrictions and produced the highest proportion of unfavourable findings [34].

More recent randomised evidence remains nuanced. Sephien et al. conducted a systematic review and meta-analysis of nine randomised trials comparing shorter and longer resident duty hours. Shorter hours were associated with small reductions in emotional exhaustion and dissatisfaction with overall wellbeing, but there was no statistically significant difference in serious medical errors or hospital length of stay [33]. The effects of working-time interventions therefore appear to depend on more than the number of hours removed from a rota.

For postgraduate education, the implication is that working-time interventions cannot be judged by hours alone. Reducing exposure may affect continuity and learning opportunities, whereas preserving exposure without addressing workload, staffing or recovery may maintain experience at the expense of learning capacity and safety. Shift duration, workload intensity, staffing, supervision, handover, rest, and recovery therefore need to be considered together.

Making OOH work a safer and more effective learning environment

Graded Autonomy and Accessible Supervision

The preceding evidence supports an approach to OOH supervision that preserves meaningful trainee responsibility while ensuring that assistance is available when required. In practical terms, this means matching initial assessment and decision-making opportunities to the trainee's developing capability, providing clear routes to senior advice and encouraging discussion of important clinical decisions afterwards [2,4,10]. Evidence concerning overnight attending availability suggests that accessible supervision need not eliminate perceived autonomy, although the effects depend on how supervision is provided [21,22].

Escalation should consequently be easy and psychologically safe while still requiring trainees to articulate their assessment, reasoning and proposed plan. In this model, supervision functions simultaneously as a safety mechanism and an educational resource.

Preparing Doctors for OOH Responsibility

Preparation can reduce avoidable uncertainty before substantial OOH responsibility is assumed. Simulation is useful because it can reproduce prioritisation, communication, deterioration management, and escalation without exposing patients to the consequences of novice error.

Existing UK simulation programmes have incorporated competing clinical tasks, handover, prioritisation, access to simulated senior support, and debriefing [14,15]. These components offer practical examples of how induction can prepare trainees for the organisation and decision-making demands of OOH work.

These individual programmes sit within a broader literature on preparation for OOH practice. Gordon et al.'s UK scoping review identified 18 educational interventions, predominantly using simulation and targeting competencies including prioritisation, communication, clinical decision-making, and leadership. Thirteen studies reported improvements in confidence or attitudes, whereas only three incorporated measurable performance outcomes and one reported behavioural change within a simulated environment; most lacked long-term follow-up [12]. Simulation should therefore be regarded as a promising method of preparing trainees for the structure and demands of OOH work rather than as evidence that preparedness training alone produces sustained clinical competence.

The educational target is therefore not simply knowledge of individual acute conditions, but the ability to decide what requires attention first, what can safely wait, what information is needed, and when assistance should be sought.

OOH induction could accordingly include deterioration management, prioritisation, structured handover, communication, escalation pathways, senior-support arrangements, and practical workflow. The aim is not to make a novice independent before clinical exposure, but to provide enough scaffolding for authentic experience to become progressively more sophisticated learning.

Educational support may also need to change with experience. Kaplan et al. reported increased self-reported confidence after a 12-module night-float curriculum for 76 first-year internal medicine residents, with the largest effects early in the academic year [37]. Although confidence is not equivalent to competence, the finding is consistent with more intensive scaffolding early in training and progressive adjustment as capability develops.

Turning Clinical Exposure Into Learning

Clinical exposure alone does not guarantee learning. OOH doctors may assess large numbers of patients and make numerous decisions, but discontinuity between night and daytime teams can mean that they do not subsequently observe a patient's clinical course or discover whether their initial diagnosis and management were appropriate. Without outcome information, reflection, or feedback, repeated exposure may generate experience without fully converting that experience into improved future practice.

This limitation has been demonstrated directly in night-float education. Lim et al. noted that residents working night float received relatively little feedback regarding diagnostic and management reasoning. Their Decisions in the Dark intervention asked residents to review the subsequent clinical course of selected patients whom they had admitted overnight, complete structured reflections, and obtain feedback from daytime attending physicians and senior residents. Sixty-eight of 82 eligible residents participated, and analysis of 248 reflection worksheets identified themes including recognition of clinical-reasoning gaps, evolution of differential diagnoses, changes residents intended to make in future practice, and reflection on interactions with other clinicians [38].

This illustrates the distinction between clinical exposure and the educational processing of that exposure. Outcome review, discussion, and reflection can convert an isolated overnight decision into information that informs future judgement.

Training programmes could therefore reconnect selected OOH decisions with subsequent outcomes through brief case review, targeted feedback after escalation, or structured reflection. Such approaches need not reproduce lengthy daytime teaching; their purpose is to close the learning loop and make experienced clinical reasoning more visible.

Night-specific educational interventions may provide additional structure. Resident-led nocturnal curricula, faculty-guided nighttime teaching, and modular night-float curricula have all been described [37,39,40]. However, these examples largely represent single-centre North American programmes and commonly evaluate resident perceptions, acceptability, or confidence rather than objective clinical competence or patient outcomes [37,39,40]. They should therefore be regarded as promising educational strategies rather than proof that formal nighttime curricula necessarily improve clinical outcomes.

Nighttime education should also fit the clinical environment. Workload, interruptions, and fatigue may make lengthy scheduled teaching impractical, whereas brief case-based discussion, bedside teaching and targeted feedback may be more compatible with OOH work [13,39,40].

Designing a Sustainable OOH Learning Environment

Educational interventions alone cannot compensate for an unsustainable working environment. As discussed in the preceding section, fatigue, demanding work patterns, and insufficient recovery can affect wellbeing and perceived learning capacity [5-7,33]. Consequently, staffing, workload, and rota design should be regarded not only as service-delivery considerations but also as educational variables.

This systems perspective is supported by Tucker et al.'s recent scoping review of OOH junior medical officer work. The review identified workload, staffing, and supervision as important features of the OOH environment and described strategies, including reducing or redistributing unnecessary OOH tasks, structured organisational approaches to handover and workflow, electronic task-management systems, and increased senior involvement [1]. From an educational perspective, however, reducing unnecessary workload should be distinguished from eliminating meaningful clinical responsibility: service redesign should preserve appropriate opportunities for assessment, prioritisation, and decision-making while reducing work that contributes little to either patient care or trainee development.

Responsibility should therefore not be treated as a dose-response intervention in which more is always better. Too little may restrict opportunities for independent judgement, whereas excessive workload and fatigue can consume the cognitive and temporal resources needed for learning.

Strategies for improving nighttime education have included interventions addressing the sleep-wake cycle, personnel and formal educational components. Weltz et al.'s review identified these as three recurring categories of strategies across 42 articles examining the educational value of night-float rotations [13]. However, the evidence reviewed throughout this article does not identify a single intervention capable of eliminating the educational and physiological consequences of night work. Fatigue management should therefore not be reduced to individual resilience or personal coping strategies.

Programmes should therefore consider shift design, staffing, workload intensity, breaks, recovery between shifts, and access to appropriate rest facilities together [5-7,32,33]. Individual measures such as sleep education or planned rest may help but cannot compensate reliably for an excessive clinical workload.

Workload also determines whether educational processes are feasible: feedback requires time, reflection requires cognitive capacity, supervision requires available seniors, and even brief teaching becomes difficult during continuous urgent work. A clinically busy environment can therefore provide abundant exposure while remaining educationally impoverished.

No single intervention is likely to create an effective OOH learning environment. Greater senior presence can constrain judgement if it removes meaningful responsibility, whereas autonomy without reliable support creates avoidable risk [4,21,22]. Likewise, reducing working hours without considering handover, continuity, workload compression, and clinical exposure may replace one problem with another [33,34].

Taken together, the evidence supports a systems approach to supported experiential learning. OOH work should provide responsibility graded to capability, accessible senior support, preparation for practical on-call demands, and opportunities for feedback and reflection. Staffing and workload should leave sufficient cognitive and temporal capacity for learning, while rota design and recovery should limit avoidable fatigue.

The zone of meaningful responsibility is proposed here as an author-developed conceptual synthesis of the literature reviewed, rather than a previously established or empirically validated educational model. It integrates experiential workplace learning, progressive independence and supervisory entrustment with evidence concerning OOH workload, fatigue, supervision, and opportunities for feedback [2-10]. Within this proposed framework, clinical responsibility creates opportunities for autonomous assessment, prioritisation, and decision-making under uncertainty; recognising personal limits and escalating appropriately enables access to more experienced clinical reasoning and collaborative management; and feedback and reflection may help translate these experiences into future capability. The framework proposes that these processes are more likely to support learning when responsibility is matched to capability, senior support remains accessible, and workload and fatigue do not overwhelm the trainee's capacity to learn.

The framework is intended as an interpretive guide for educational design, not a validated predictor of trainee or patient outcomes. Its boundaries have not been empirically measured, and the relationships proposed should not be interpreted as established causal effects. For postgraduate training, the implication is not to eliminate challenge or maximise independence, but to provide meaningful responsibility alongside appropriate supervision, opportunities for feedback, adequate staffing, and sufficient recovery. These proposed relationships and the conditions that may support or disrupt them are summarised in Figure 1.

**Figure 1:**
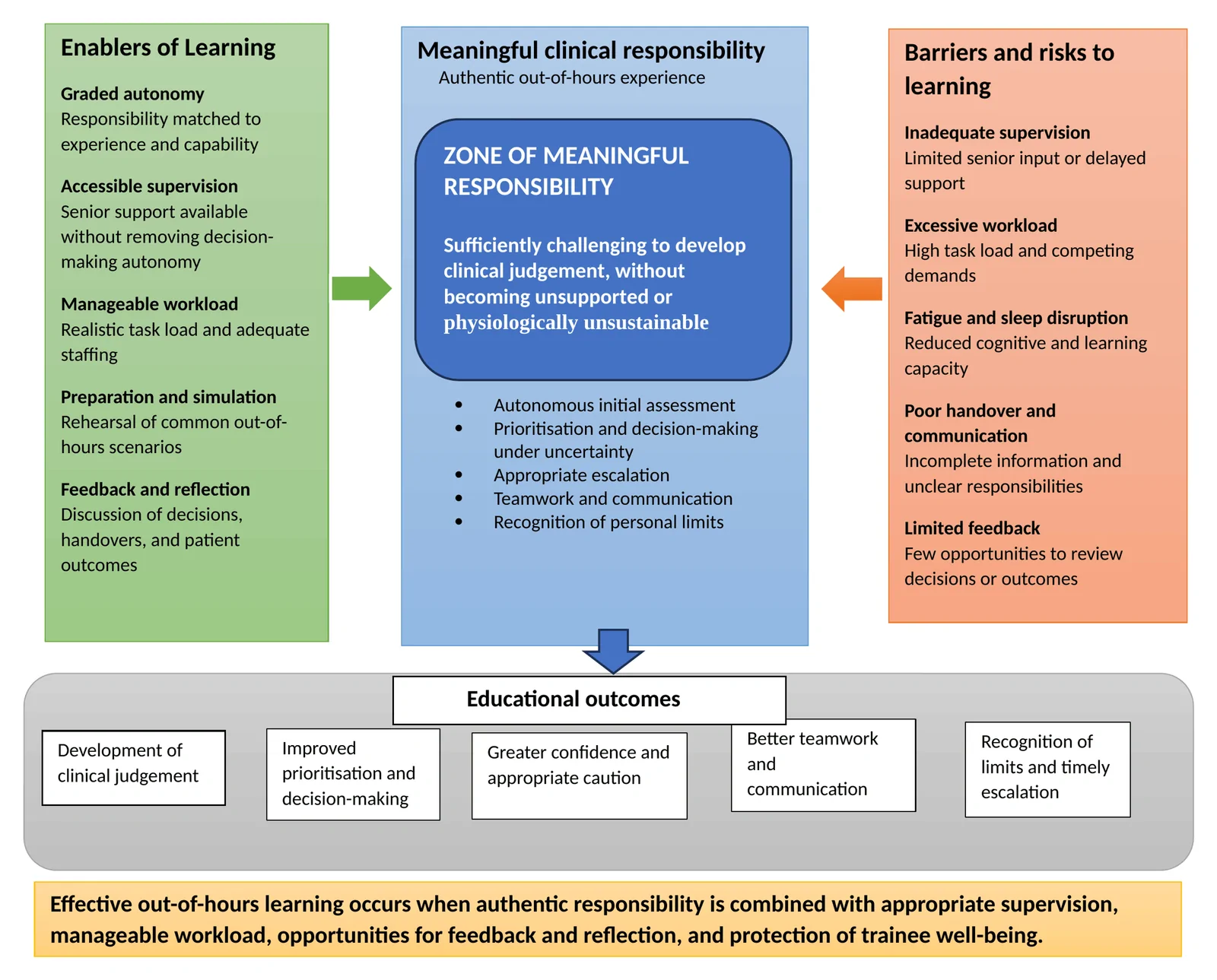
The Zone of Meaningful Responsibility for Out-of-Hours Learning in Junior Doctor Training The figure presents an author-developed conceptual synthesis informed by experiential workplace learning, progressive independence, and supervisory entrustment, integrated with evidence concerning out-of-hours workload, fatigue, supervision, and feedback. It illustrates the proposed relationship between meaningful clinical responsibility, the conditions that support or disrupt learning, and educational development. The framework has not been empirically validated, and the relationships shown should not be interpreted as established causal effects or measured thresholds. This is an original figure developed by the author; all concepts and content are original, and no third-party or previously published media were used. The figure was created in Microsoft Word (Microsoft Corporation, Redmond, Washington) using basic shapes, arrows, text boxes, and colours. ChatGPT (OpenAI, San Francisco, California) was used to assist with the visual layout/formatting and preparation of the final PNG to meet the journal figure specifications.

## Conclusions

OOH work can be a valuable learning environment for junior doctors when authentic clinical responsibility is matched by appropriate support. Its educational value lies in opportunities to assess patients, prioritise competing demands, make decisions under uncertainty, recognise personal limits, escalate appropriately, and learn from subsequent feedback and reflection. However, responsibility is not inherently educational: when autonomy exceeds capability or available supervision, or when workload and fatigue overwhelm the capacity for safe reasoning and learning, the same environment can become educationally and clinically counterproductive.

Postgraduate training should therefore aim to maintain junior doctors within a zone of meaningful responsibility, avoiding both unsupported practice and physiologically unsustainable demands. This requires graded autonomy, accessible senior supervision, preparation for OOH demands, manageable workload, opportunities for feedback and reflection, and sufficient rest and recovery. The objective should not be to remove the challenge from OOH work but to create the conditions in which that challenge remains safe, supported, and educationally productive.
